# Healthcare service provided for headache patients in selected African countries: a cross-sectional survey of clinician perspectives

**DOI:** 10.3389/fneur.2026.1853307

**Published:** 2026-07-15

**Authors:** Amr Hassan, Freda Dodd-Glover, Najib Kissani, Magnerou Annick Melanie, Sounga Bandzouzi Prince Eliot Galieni, Mendinatou Agbetou, Kigocha Okeng'o, Daniel Gams Massi, Evelyne Diarra, Agbo Panzo Cedric, Chiamaka Edith Okereke, Wael Alwachi, Osheik Seidi, Kadira Abdi Aden, Augustina Charway-Felli, Emna Ellouz, Foksouna Sakadi, John N. Jabang, Denis Shatima, Athanase Millogo, Ahmed Allioueche, Fatimata Hassane Djibo, Tiwonge Elisa Phiri, Jamal Barros Baco, Grenaba-Duval Lewis Reinier Joël, Frighton Mutete, Doaa M. Khalil, Mona Hussein

**Affiliations:** 1 Department of Neurology, Cairo University, Cairo, Egypt; 2 Neurology Unit, Department of Medicine and Therapeutics, Korle Bu Teaching Hospital, Accra, Ghana; 3 Department of Neurology, Cadi Ayyad University, Marrakech, Morocco; 4 Faculty of Medicine and Pharmaceutical Sciences, University of Douala, Douala, Cameroon; 5 Loandjili General Hospital, Pointe-Noire, Republic of Congo; 6 Faculty of Health Sciences, Marien Ngouabi University, Brazzaville, Republic of Congo; 7 Teaching Hospital of Borgou/Health Faculty of University of Parakou, Parakou, Benin; 8 Muhimbili National Hospital, Dar es Salaam, Tanzania; 9 Faculty of Medicine and Pharmaceutical Sciences, University of Doula, Douala, Cameroon; 10 Department of Neurology, CHU Cocody, University Felix Houphouet, Boigny-Abidjan, Cote d’Ivoire; 11 University of Nigeria Teaching Hospital, Enugu, Nigeria; 12 Department of Neurology, Ali Omar Askar Hospital, Tripoli, Libya; 13 Faculty of Medicine, University of Khartoum, Khartoum, Sudan; 14 Hôpital Militaire de Djibouti, Djibouti, Djibouti; 15 37 Military Hospital, Accra, Ghana; 16 Department of Neurology, Gabes Hospital, Sfax University, Sfax, Tunisia; 17 Department of Neurology, Nationale Reference Teaching Hospital, N'Djamena, Chad; 18 Edward Francis Small Teaching Hospital, University of Gambia, Serekunda, Gambia; 19 National Hospital Abuja, Abuja, Nigeria; 20 Centre Hospitalier Universitaire, Souro Sanou, Bobo-Dioulasso, Burkina Faso; 21 CHU, Constantine, Algeria; 22 Department of Neurology, Hospital National Amirou Boubacar Diallo of Niamey, Niamey, Niger; 23 Queen Elizabeth Central Hospital, Blantyre, Malawi; 24 Hospital Central da Beira, Beira, Mozambique; 25 Université de Bangui, Bangui, Central African Republic; 26 Livingstone University Teaching Hospital, Livingstone, Zambia; 27 Public Health and Community Medicine, Beni-Suef University, Beni Suef, Egypt; 28 Department of Neurology, Beni-Suef University, Beni-Suef, Egypt

**Keywords:** Africa, headache, healthcare service, insurance coverage, low-income countries

## Abstract

**Background:**

Headache disorders represent a major global health burden that is highly prevalent across the continent of Africa; however, they remain underdiagnosed and inadequately managed. The objective of this study was to evaluate the availability, accessibility, and organizational structure of headache care services across African countries and to detect major challenges and barriers to optimal care from the perspective of headache-treating clinicians.

**Methods:**

Countries were chosen using a stratified sampling strategy based on World Bank income classification [low-income countries (LICs), lower-middle-income countries (LMICs), and upper-middle-income countries (UMICs)] to ensure balanced representation across economic strata. Within each stratum, eligible neurologists were identified through national neurology societies and indexed publications. Of 120 invited clinicians from 40 countries, 73 neurologists from 28 countries responded (response rate 60.8%). Participants were invited to fill out a structured online questionnaire to assess the healthcare system structure (including workforce capacity, healthcare system characteristics, and diagnostic infrastructure), availability of the essential and newer headache medications, and perceived barriers to optimal care in their countries.

**Results:**

Significant discrepancies were observed in workforce capacity, defined as the reported number of neurologists per country ranging from 10 (IQR: 4.75–34.5) in LICs to 200 (IQR: 97–700) in UMICs (*p* < 0.001). Access to headache care was limited in LICs, where 50.0% (6/12) of respondents reported a 3–4 week waiting list for elective neurological consultations, while 41.7% (5/12) reported a similar 3–4 week waiting time for imaging (*p* = 0.028, 0.012). Self-medication was universal (100%). Structural barriers were highest in LICs, including limited neuroimaging (91.7%) and financial constraints (83.3%). Essential medications (according to WHO (World Health Organization) and International Headache Society (IHS) practice recommendations) were relatively more reimbursed in LMICs/UMICs; however, innovative therapies (gepants, ditans, and calcitonin gene-related peptide (CGRP) mAbs) were not reimbursed across all settings.

**Conclusion:**

Headache care across African countries is facing substantial inequities in human resources, workforce capabilities, and access to proper care. Addressing these challenges requires fostering the healthcare systems, training of non-specialist healthcare providers, expanding insurance coverage, and increasing awareness of headache disorders in health policy to enhance patient outcomes.

## Introduction

Headache disorders constitute a significant global public health concern. They include primary headache syndromes such as migraine, tension-type headache, and cluster headache, as well as secondary headache disorders. According to the Global Burden of Disease Study 2019, headache disorders are among the leading causes of disability worldwide, contributing substantially to global years lived with disability (YLDs). Migraine was the second leading cause of YLDs, accounting for approximately 45.1 million YLDs, while tension-type headache ranked third, contributing about 7–8 million YLDs (1). In African countries particularly those in sub-Saharan region, headache disorders are highly prevalent yet historically understudied. Epidemiological data from Benin showed a notably high lifetime prevalence of primary headache disorders, reaching up to 95.2% (2, 3). In Cameroon, a community-based study demonstrated an exceptionally high headache burden, with lifetime prevalence of headache reaching 94.8% and one-year prevalence reported at 77% (4). In other African countries such as Zambia, the adjusted 1-year prevalence of any headache was found to be 61.6% (5), whereas in Ethiopia, it was 44.9% (6).

Despite this high epidemiological burden of headache across African countries, it remains underdiagnosed and inadequately managed, particularly in low- and middle-income countries where trained specialists are scarce and access to essential diagnostic and therapeutic resources is limited (7, 8). It worth noting that the low levels of health literacy, widespread self-medications, and reliance on traditional remedies, further contributes to delays in diagnosis and treatment of headache in these countries (9).

There is a marked paucity of data on the quality of headache care across African countries, limiting insight into the landscape of headache management in these countries. Existing literature has primarily shed light on the prevalence and burden of headache disorders, without paying considerable attention to workforce capacity, availability and accessibility of different diagnostic and therapeutic modalities, challenges and barriers to effective headache care. This gap in providing reliable data about headache care in African countries hinders the identification of shortcomings in healthcare system, undermines the optimal allocation of healthcare resources, and hampers the development of evidence-based strategies to improve headache diagnosis and management (10, 11). Without robust country-specific data, efforts to advance effective headache care policies across African countries will remain limited, thereby perpetuating inequities in patient access to diagnostic and treatment services.

The objective of this work is to evaluate the structure, availability, and accessibility of healthcare services for patients with headache disorders across African countries based on insights provided by headache specialists. The second objective was to identify challenges and barriers to delivering effective headache care from the perspective of these specialists. Gathering specialists’ opinions in this way is expected to be crucial for guiding strategies to improve headache care across African populations.

## Methods

### Study design

This cross-sectional study was conducted on 73 neurologists from 28 African countries. Recruitment was done from October 1, 2025, to January 1, 2026. The study was conducted in accordance with the Strengthening the Reporting of Observational Studies in Epidemiology (STROBE) guidelines for observational studies (12).

### Eligibility criteria

**Figure 1:**
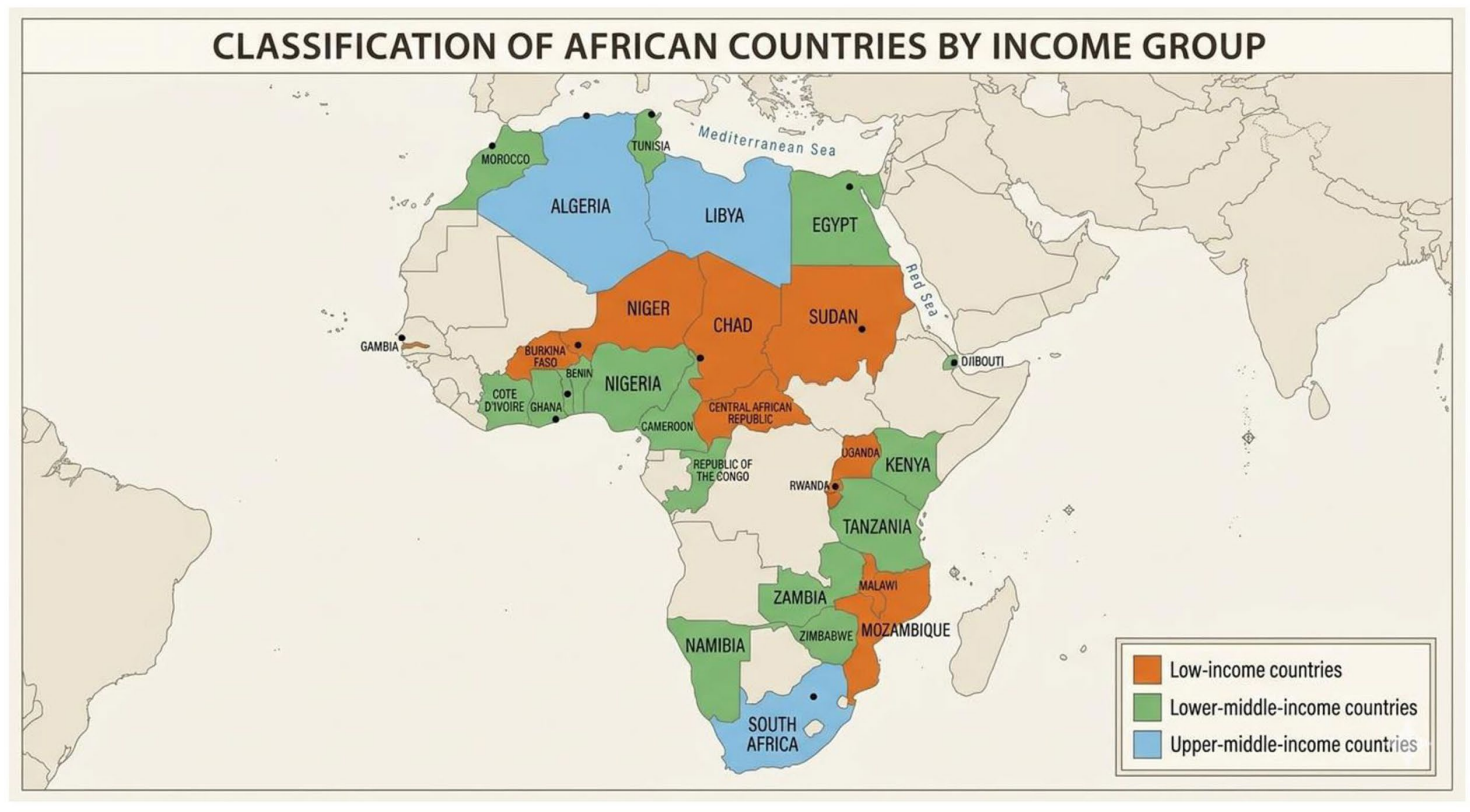
Geographic distribution of participating countries across Africa, categorized according to World Bank income classification. This figure was generated by the authors using AI-assisted visualization tools based on publicly available World Bank data. No copyright permission was required.

The study included African neurologists with recognized expertise in headache medicine. This was defined based on active clinical involvement in headache management and documented professional experience, including at least 5 years of headache practice and/or professional involvement in neurology/headache societies or related academic activities. The following African countries were included: Burkina Faso, Central African Republic, Chad, Gambia, Malawi, Mozambique, Niger, Rwanda, Sudan, Uganda, Egypt, Tunisia, Morocco, Ghana, Cameroon, Cote d’Ivoire, Benin, Kenya, Djibouti, Nigeria, Namibia, Republic of the Congo, Tanzania, Zambia, Zimbabwe, Algeria, Libya, and South Africa. The included countries were classified according to income based on the World Bank income classification system into low-income countries (LICs), lower-middle-income countries (LMICs), and upper-middle-income countries (UMICs) (13) (Figure 1).

Non-African clinicians were excluded to ensure that survey responses reflected the perspectives of specialists trained and socialized within African medical systems. Also, African clinicians who are not currently practicing in or affiliated with healthcare institutions in African countries were excluded.

### Survey development

The survey was developed through a collaboration between neurologists from different African countries in accordance with the Checklist for Reporting Results of Internet E Surveys (CHERRIES) guidelines (14). These neurologists should have recognized expertise in headache medicine, based on their professional qualifications and clinical practice. Specifically, the clinicians involved in survey development were senior neurologists with at least 5 years of active clinical experience in headache management, and many held leadership roles in national or regional neurology societies. Initially, the neurologists outlined the key domains that should be thoroughly evaluated to provide a comprehensive overview of the shortcomings of the healthcare service provided to headache patients in African countries. Following this step, the neurologists collaboratively developed a draft questionnaire addressing the predefined key domains. This questionnaire included 25 open-and closed-ended questions. The draft questionnaire was developed in English and underwent several rounds of iterative review by the selected neurologists. These revisions focused on clarity, cultural appropriateness, and alignment with study objectives. Although no external linguistic expert was engaged, the multidisciplinary team’s collective experience in multinational research was deemed sufficient to ensure suitability for dissemination.

To further refine the questions included in the survey, a pilot testing phase was conducted on 10 neurologists from different African countries. During this phase, the neurologists were engaged in structured online meetings conducted by the research team. The study objectives were explained, and participants were asked to complete the draft survey. Their feedback was collected both verbally during the meetings and subsequently confirmed through written communication (email). Their input was used to further optimize the clarity and relevance of the survey and resolve any potential ambiguities or misunderstanding.

The final refined survey consisted of 18 questions covering the following key domains (Supplementary material 1):

1. Demographics and professional profiles of the included participants
2. Characteristics of healthcare system
3. Availability of medications and other therapeutic modalities for headache management
4. Challenges to provide effective healthcare for headache patients

### Data collection

The survey was developed on Google Form to facilitate efficient data collection (Google LLC) (15). Google Form was sent electronically to the participants via professional email networks. Each email also included a brief overview of the study objectives, explanation of what is required from each participant, and a consent statement. Participants were instructed to consult the relevant healthcare authorities or official reference sources, whenever clarification was required, to verify the accuracy and reliability of the information provided.

To maximize the response rate, two reminder emails were sent at two-week intervals. The research team regularly checked submissions to exclude any duplicate or incomplete responses from the final dataset.

Survey responses were stored on encrypted servers. Access to the dataset was restricted to the principal investigator and authorized members of the research team through password-protected institutional accounts. Data were subsequently downloaded into encrypted files for statistical analysis, ensuring confidentiality and secure handling throughout the study.

Variables were analyzed at two levels: clinician-level (*n =* 73) for practice-related responses, and country-level (*n =* 28) for national workforce indicators. Workforce size was analyzed as reported absolute numbers, without normalization by population size, due to lack of consistent denominators across all 28 countries.

### Outcomes

The primary outcome was to provide an in-depth overview of the availability, accessibility, and organization of headache care services across African countries. The secondary outcome was to characterize challenges and barriers to effective healthcare service for headache patients from clinicians` perspective.

### Ethical consideration

The study participants signed informed consent electronically, after clarifying the study aim and the importance of the study before data collection. Ethical approval was obtained from Research Ethics committee, Faculty of medicine, Beni-Suef University. Provisional approval to initiate recruitment was granted in September 2025, authorizing the start of participants enrollment. The formal approval certificate was subsequently issued on December 2, 2025 (Approval number: FMBSUREC/02122025/Khalil).

### Sampling

The 54 African countries were categorized into LICs, LMICs, and UMICs based on World Bank classification (13). A stratified sampling strategy was employed at the country level, based on World Bank income classification to ensure balanced representation across economic strata. Within each stratum, neurologists were identified through national and regional professional societies as well as peer-reviewed publications. Eligibility was restricted to neurologists with recognized expertise in headache medicine, defined by active clinical practice and documented professional involvement (e.g., membership in national neurology/headache societies or authorship of peer-reviewed publications). Participants were identified by the study authors through professional societies and indexed publications. A total of 120 clinicians from 40 African countries in the three income categories were invited to participate in the survey. Of them, 73 neurologists (from 28 countries) responded, yielding a response rate of 60.8%.

### Statistical analysis

IBM SPSS (Statistical Package of Social Science) Version 25 was used to analyze the data. Kolmogorov–Smirnov test was used to test the normality of data. Categorical variables were expressed as numbers and percentages. Quantitative variables were expressed as median and inter quartile range (IQR). For comparisons across income groups, we applied the Kruskal–Wallis test and Chi-squared test to the appropriate unit of analysis (country-level data for workforce size; clinician-level data for practice-related variables). *p* < 0.05 was considered statistically significant. All tests were two-tailed.

## Results

### Demographics and professional profiles of the included participants

This cross-sectional study was conducted on 73 neurologists. Participants represented 28 countries across the three World Bank income categories. LICs included Burkina Faso, Central African Republic, Chad, Gambia, Malawi, Mozambique, Niger, Rwanda, Sudan, and Uganda. LMICs included Egypt, Tunisia, Morocco, Ghana, Cameroon, Cote d’Ivoire, Benin, Kenya, Djibouti, Nigeria, Namibia, Republic of the Congo, Tanzania, Zambia, and Zimbabwe. UMICs included Algeria, Libya, and South Africa.

The median age of the participants was 43 (38–52) years. The majority were males 43/73 (58.9%), while females represented 30/73 (41.1%) of the participants. Regarding geographical distribution, most participants were from LMICs 41/73 (56.2%), followed by UMICs 20/73 (27.4%) and LICs 12/73 (16.4%). In terms of primary workplace, more than half of the participants worked in university hospitals 41/73 (56.2%), followed by general hospitals 18/73 (24.7%). Smaller proportions were employed in private clinics 6/73 (8.2%), insurance hospitals 5/73 (6.8%), and private hospitals 3/73 (4.1%). The median duration of experience in headache practice was 10 (7–21.5) years (Table 1).

**Table 1:** Demographics and professional profiles of the included neurologists.

Variables		Participants (*n =* 73)
Age [median (IQR)]		43 (38–52)
Sex [*n* (%)]	Males	43 (58.9%)
	Females	30 (41.1%)
Country [*n* (%)]	LICs^1^	12 (16.4%)
	LMICs ^2^	41 (56.2%)
	UMICs ^3^	20 (27.4%)
Primary workplace [*n* (%)]	General hospital^4^	18 (24.7%)
	Insurance hospital^5^	5 (6.8%)
	University hospital	41 (56.2%)
	Private hospital^6^	3 (4.1%)
	Private clinic^7^	6 (8.2%)
Years of experience in Headache [median (IQR)]		10 (7–21.5)

LICs, Low-income countries; LMICs, Lower-middle-income countries; UMICs, upper-middle-income countries. ^1^Burkina Faso, Central African Republic, Chad, Gambia, Malawi, Mozambique, Niger, Rwanda, Sudan, Uganda. ^2^Egypt, Tunisia, Morocco, Ghana, Cameroon, Cote d’Ivoire, Benin, Kenya, Djibouti, Nigeria, Namibia, Republic of the Congo, Tanzania, Zambia, Zimbabwe. ^3^Algeria, Libya, South Africa. ^4^Public sector hospitals providing broad medical services, typically government-funded and serving the general population. ^5^Hospitals affiliated with national or social insurance schemes, where access to services is linked to insurance coverage. ^6^Hospitals owned and operated by private entities, where patients pay directly or through private insurance. ^7^Outpatient facilities that are independently owned and operated by physicians or private entities.

### Healthcare system and human resources

The estimated number of neurologists per country differed significantly between the three groups, with a median of 10 (4.75–34.5) in LICs, 27 (15.25–233.75) in LMICs, and 200 (97–700) in UMICs (*p <* 0.001). Similarly, there was a significantly higher number of headache clinics in UMICs compared to LMICs and LICs (*p <* 0.001) (Table 2).

**Table 2:** Healthcare system and human resources.

Variables		LICs (*n =* 12)	LMICs (*n =* 41)	UMICs (*n =* 20)	*P*-value
The estimated number of neurologists per country [median (IQR)]		10 (4.75–34.5)	27 (15.25–233.75)	200 (97–700)	<0.001*
The estimated number of headache clinics [*n* (%)]	0	5 (41.7%)	15 (36.6%)	0	<0.001*
	1-5	4 (33.3%)	14 (34.1%)	0	
	6–10	2 (16.7%)	6 (14.6%)	4 (20.0%)	
	11–15	1 (8.3%)	2 (4.9%)	6 (30.0%)	
	More than 15	0	4 (9.8%)	10 (50.0%)	
Healthcare providers other than neurologists who diagnose and treat headaches [*n* (%)]	General practitioners	12 (100.0%)	38 (92.7%)	18 (90.0%)	0.547
	Internal medicine doctors	11 (91.7%)	40 (97.6%)	17 (85.0%)	0.185
	Nurses	7 (58.3%)	8 (19.5%)	1 (5.0%)	0.002*
	Pharmacists	7 (58.3%)	20 (48.8%)	2 (10%)	0.005*
Medical guidelines followed in headache management [*n* (%)]	IHS	9 (75.0%)	37 (90.2%)	14 (70.0%)	0.118
	EHF	1 (8.3%)	4 (9.8%)	1 (5.0%)	0.817
	AHS	2 (16.7%)	12 (29.3%)	5 (25.0%)	0.677
	NICE	3 (25.0%)	12 (29.3%)	5 (25.0%)	0.921
	Local/Regional guidelines	5 (41.7%)	14 (34.1%)	8 (40.0%)	0.847
The average waiting list for headache patients to get elective neurological consultation [*n* (%)]	No waiting list	0	14 (34.1%)	8 (40.0%)	0.028*
	Less than 1 week	0	10 (24.4%)	4 (20.0%)	
	1–2 weeks	2 (16.7%)	7 (17.1%)	5 (25.0%)	
	3–4 weeks	6 (50.0%)	6 (14.6%)	3 (15.0%)	
	5–6 weeks	2 (16.7%)	1 (2.4%)	0	
	7–8 weeks	1 (8.3%)	1 (2.4%)	0	
	More than 8 weeks	1 (8.3%)	2 (4.9%)	0	
The average waiting list for headache patients to do investigations [*n* (%)]	No waiting list	0	11 (26.8%)	7 (35.0%)	0.012*
	Less than 1 week	0	8 (19.5%)	2 (10%)	
	1–2 weeks	3 (25.0%)	9 (22.0%)	5 (25.0%)	
	3–4 weeks	5 (41.7%)	9 (22.0%)	6 (30.0%)	
	5–6 weeks	3 (25.0%)	0	0	
	7–8 weeks	0	1 (2.4%)	0	
	More than 8 weeks	1 (8.3%)	3 (7.3%)	0	
Type of Telemedicine available for headache patients [*n* (%)]	Local Telemedicine	6 (50.0%)	28 (68.3%)	10 (50.0%)	0.285
	Regional Telemedicine	3 (25.0%)	12 (29.3%)	7 (35.0%)	0.823
	International Telemedicine	3 (25.0%)	10 (24.4%)	2 (10%)	0.391

LICs, Low-income countries; LMICs, Lower-middle-income countries; UMICs, upper-middle-income countries; AHS, American Headache Society; EHF, European Headache Federation; IHS, International Headache Society; NICE, National Institute for Health and Care Excellence. ^*^p < 0.05 is considered significant.

General practitioners were the most commonly involved healthcare providers in headache management across the three groups [12/12 (100.0%), 38/41 (92.7%), and 18/20 (90.0%) in LICs, LMICs, and UMICs, respectively], with no significant difference between groups. Internal medicine physicians were also widely involved. However, the involvement of nurses and pharmacists differed significantly, being more common in LICs 7/12 (58.3%) for both compared to LMICs 8/41 (19.5%) and 20/41 (48.8%), and UMICs 1/20 (5.0%) and 2/20 (10.0%), respectively (*p =* 0.002 and 0.005, respectively) (Table 2).

There were no statistically significant differences between groups regarding the medical guidelines used for headache management, with the International Headache Society (IHS) guidelines being the most commonly followed across all the three groups (Table 2).

Regarding access to care, waiting times for elective neurological consultation differed significantly between the three groups (*p* = 0.028). Half of LICs 6/12 (50.0%) reported waiting times of 3–4 weeks, whereas a notable proportion of LMICs and UMICs reported either no waiting list 14/41 (34.1%) and 8/20 (40.0%), respectively, or waiting times of less than 1 week 10/41 (24.4%) and 4/20 (20.0%), respectively. Similarly, waiting times for investigations were significantly longer in LICs (*p =* 0.012), with 5/12 (41.7%) of patients waiting 3–4 weeks. Conversely, 11/41 (26.8%) of participants from LMICs and 7/20 (35.0%) from UMICs reported no waiting list for investigations. These investigations included brain imaging [computed tomography (CT) and magnetic resonance imaging (MRI)], laboratory tests (basic blood work, metabolic profile), and specialized procedures such as lumbar puncture (Table 2).

The availability of telemedicine services did not differ significantly across the three groups. Local telemedicine was the most common type reported, followed by regional and international telemedicine services (Table 2).

### Reimbursement of medications and other therapeutic modalities for headache management

Overall, reimbursement of acute migraine medications was higher in LMICs and UMICs than in LICs. Among analgesics, diclofenac showed a significant difference across groups (*p =* 0.023), with higher reimbursement in LMICs and UMICs. Similarly, the reimbursement of antiemetics such as metoclopramide and domperidone was significantly greater in UMICs (*p =* 0.035 and 0.013, respectively). Although triptans were more frequently reimbursed in UMICs and LMICs, these differences did not reach statistical significance. Newer agents, including gepants and ditans, were not reimbursed across all three groups (Table 3).

**Table 3:** Reimbursement of medications and other therapeutic modalities for headache management.

Variables		LICs (*n =* 12)	LMICs (*n =* 41)	UMICs (*n =* 20)	*P*-value
Reimbursed acute migraine medications [*n* (%)]	Paracetamol	6 (50.0%)	32 (78.0%)	17 (85.0%)	0.070
	Aspirin	6 (50.0%)	30 (73.2%)	17 (85.0%)	0.099
	Ibuprofen	6 (50.0%)	32 (78.0%)	14 (70.0%)	0.167
	Naproxen	3 (25.0%)	21 (51.2%)	12 (60.0%)	0.149
	Diclofenac	5 (41.7%)	31 (75.6%)	17 (85.0%)	0.023*
	Indomethacin	4 (33.3%)	20 (48.8%)	13 (65.0%)	0.208
	Sumatriptan	1 (8.3%)	10 (24.4%)	9 (45.0%)	0.064
	Rizatriptan	0	4 (9.8%)	1 (5.0%)	0.465
	Zolmitriptan	2 (16.7%)	5 (12.2%)	4 (20.0%)	0.716
	Naratriptan	1 (8.3%)	1 (2.4%)	1 (5.0%)	0.646
	Almotriptan	1 (8.3%)	0	1 (5.0%)	0.229
	Eletriptan	0	3 (7.3%)	0	0.295
	Frovatriptan	0	1 (2.4%)	0	0.673
	Ergotamine tartrate	2 (16.7%)	14 (34.1%)	5 (25.0%)	0.455
	Dihydroergotamine	1 (8.3%)	15 (36.6%)	8 (40.0%)	0.136
	Metoclopramide	5 (41.7%)	29 (70.7%)	17 (85.0%)	0.035*
	Domperidone	2 (16.7%)	19 (46.3%)	14 (70.0%)	0.013*
	Prochlorperazine	2 (16.7%)	13 (31.7%)	6 (30.0%)	0.593
	Chlorpromazine	4 (33.3%)	22 (53.7%)	9 (45.0%)	0.442
	Ubrogepant	0	0	0	-
	Rimegepant	1 (8.3%)	1 (2.4%)	0	0.370
	Zavegepant	1 (8.3%)	0	1 (5.0%)	0.229
	Lasmiditan	0	0	0	-
Reimbursed preventive migraine medications [*n* (%)]	Propranolol	6 (50.0%)	32 (78.0%)	17 (85.0%)	0.070
	Metoprolol	2 (16.7%)	25 (61.0%)	11 (55.0%)	0.025*
	Atenolol	6 (50.0%)	28 (68.3%)	12 (60.0%)	0.487
	Nadolol	3 (25.0%)	7 (17.1%)	0	0.088
	Amitriptyline	6 (50.0%)	28 (68.3%)	17 (85.0%)	0.107
	Nortriptyline	3 (25.0%)	8 (19.5%)	0	0.078
	Venlafaxine	2 (16.7%)	13 (31.7%)	10 (50.0%)	0.137
	Valproic acid	6 (50.0%)	29 (70.7%)	17 (85.0%)	0.106
	Topiramate	4 (33.3%)	20 (48.8%)	14 (70.0%)	0.109
	Gabapentin	4 (33.3%)	21 (51.2%)	15 (75.0%)	0.057
	Verapamil	3 (25.0%)	17 (41.5%)	8 (40.0%)	0.578
	Flunarizine	5 (41.7%)	7 (17.1%)	2 (10.0%)	0.077
	Cinnarizine	4 (33.3%)	12 (29.3%)	2 (10.0%)	0.195
	Erenumab	0	0	0	-
	Fremanezumab	0	0	0	-
	Galcanezumab	0	0	0	-
	Eptinezumab	0	0	0	-
Available interventional pain therapy for headache patients [*n* (%)]	Onabotulinumtoxin A	2 (16.7%)	27 (65.9%)	8 (40.0%)	0.006*
	Nerve block	2 (16.7%)	23 (56.1%)	5 (25.0%)	0.012*
	Radio-frequency	0	6 (14.6%)	1 (5.0%)	0.227
	Neuromodulation Techniques	1 (8.3%)	7 (17.1%)	4 (20.0%)	0.680
Available supportive therapy for headache patients [*n* (%)]	Psychological support	8 (66.7%)	35 (85.4%)	17 (85.0%)	0.306
	Physiotherapy	9 (75.0%)	27 (67.5%)	16 (84.2%)	0.395
	Nutritional counseling	6 (50.0%)	25 (61.0%)	17 (85.0%)	0.081

LICs, Low-income countries; LMICs, Lower-middle-income countries; UMICs, upper-middle-income countries. *P < 0.05 is considered significant.

Preventive migraine medications were categorized according to evidence-based efficacy levels based on current international migraine prevention guidelines, including the IHS global practice recommendations for preventive pharmacological treatment of migraine (16). Class I agents included propranolol, metoprolol, atenolol, amitriptyline, nortriptyline, topiramate, and valproic acid. Class II agents included venlafaxine, verapamil, flunarizine, and cinnarizine. Class III included Gabapentin, reflecting limited or insufficient evidence for migraine prevention. Monoclonal antibodies targeting calcitonin gene-related peptide (CGRP) were considered high-efficacy preventive therapies supported by Level A evidence in current international guidelines.

In the present study, beta-blockers, antidepressants, and antiepileptics, were more commonly reimbursed in LMICs and UMICs. A statistically significant difference was observed for metoprolol (*p =* 0.025), which was more available in LMICs and UMICs. In contrast, monoclonal antibodies targeting CGRP were not reimbursed in any of the included countries (Table 3).

Interventional pain therapies demonstrated significant disparities. OnabotulinumtoxinA was reported in 2/12 (16.7%) of LICs, 27/41 (65.9%) of LMICs, and 8/20 (40.0%) of UMICs, with a statistically significant difference (*p =* 0.006). Nerve blocks were available in 2/12 (16.7%) of LICs, 23/41 (56.1%) of LMICs, and 5/20 (25.0%) of UMICs (*p =* 0.012). Radio-frequency procedures were rarely reported 0/12 (0.0%) in LICs, 6/41 (14.6%) in LMICs, and 1/20 (5.0%) in UMICs (*p* = 0.227). Neuromodulation techniques were available in 1/12 (8.3%) of LICs, 7/41 (17.1%) of LMICs, and 4/20 (20.0%) of UMICs, with no significant difference across groups (*p =* 0.680) (Table 3). Supportive therapies, including psychological support, physiotherapy, and nutritional counseling, were generally available across the three groups, with no statistically significant differences observed (Table 3).

### Challenges and barriers to effective headache care

Several factors were identified by the included neurologists as contributing to delays in the diagnosis of certain headache cases. Cultural beliefs attributing headaches to stress, spirits, or lifestyle rather than neurological disorders were cited by 66.7% of respondents from LICs, 85.4% from LMICs, and 80.0% from UMICs (*p =* 0.349). Self-medication with analgesics was universally reported across all income groups 73/73 (100.0%). Use of traditional remedies was common, particularly in LICs 12/12 (100.0%) and LMICs 40/41 (97.6%), but less frequent in UMICs 16/20 (80.0%), with a significant difference (*p* = 0.023). Limited awareness and health literacy were noted by 11/12 (91.7%) of LICs, 32/41 (78.0%) of LMICs, and 13/20 (65.0%) of UMICs (*p* = 0.214). Wrong information from social media was reported by 5/12 (41.7%) of LICs, 32/41 (78.0%) of LMICs, and 12/20 (60.0%) of UMICs (*p =* 0.045) (Table 4).

**Table 4:** Challenges and barriers to providing effective healthcare for headache patients.

Variables		LICs (*n =* 12)	LMICs (*n =* 41)	UMICs (*n =* 20)	*P*-value
The reasons for the delay in diagnosis in some headache cases [*n* (%)]	Cultural beliefs often attribute headaches to stress, spirits, or lifestyle rather than a neurological disorder	8 (66.7%)	35 (85.4%)	16 (80.0%)	0.349
	Self-medication with analgesics	12 (100.0%)	41 (100.0%)	20 (100.0%)	–
	Use of traditional remedies	12 (100.0%)	40 (97.6%)	16 (80.0%)	0.023*
	Limited awareness and health literacy	11 (91.7%)	32 (78.0%)	13 (65.0%)	0.214
	Wrong information from social media or online sources	5 (41.7%)	32 (78.0%)	12 (60.0%)	0.045*
	Long waiting list to receive consultation with a headache specialist or to undergo investigations.	9 (75.0%)	16 (39.0%)	4 (20.0%)	0.009*
	Limited access to neuroimaging	11 (91.7%)	25 (61.0%)	6 (30.0%)	0.002*
	Out-of-pocket costs and lack of insurance	10 (83.3%)	30 (73.2%)	4 (20.0%)	<0.001*
	Overlap with other medical disorders	7 (58.3%)	35 (85.4%)	14 (70.0%)	0.106
	Limited training in headache diagnosis	11 (91.7%)	31 (75.6%)	11 (55.0%)	0.064
The main challenges in your country in providing good care for headache patients [*n* (%)]	Lack of specialized headache clinics or headache specialists	12 (100.0%)	35 (85.4%)	7 (35.0%)	<0.001*
	Insufficient insurance coverage for investigations and advanced therapies.	11 (91.7%)	37 (90.2%)	13 (65.0%)	0.031*
	Unavailability of newer therapies	12 (100.0%)	37 (90.2%)	18 (90.0%)	0.525
	Poor adherence of the patients to the prescribed medications	7 (58.3%)	30 (73.2%)	9 (45.0%)	0.095
	Overuse of over-the-counter analgesics leads to medication-overuse headache	11 (91.7%)	38 (92.7%)	19 (95.0%)	0.922
	High prevalence of comorbid anxiety, depression, or sleep disorders	7 (58.3%)	30 (73.2%)	17 (85.0%)	0.246
	Use of unregulated complementary or alternative treatments without medical guidance	11 (91.7%)	32 (78.0%)	16 (80.0%)	0.570
	Absence of local or regional guidelines tailored to African healthcare systems	12 (100.0%)	36 (87.8%)	16 (80.0%)	0.249

LICs, Low-income countries; LMICs, Lower-middle-income countries; UMICs, upper-middle-income countries. *p < 0.05 is considered significant.

Structural barriers showed significant disparities. Long waiting times for specialist consultation or investigations were more frequently reported in LICs 9/12 (75.0%) compared to LMICs 16/41 (39.0%) and UMICs 4/20 (20.0%) (*p =* 0.009). Similarly, limited access to neuroimaging (CT and MRI brain) was significantly more common in LICs 11/12 (91.7%) than in LMICs 25/41 (61.0%) and UMICs 6/20 (30.0%) (*p =* 0.002). Financial barriers, including out-of-pocket costs and lack of insurance, were also significantly higher in LICs 10/12 (83.3%) compared to LMICs 30/41 (73.2%) and UMICs 4/20 (20.0%) (*p <* 0.001). The limited awareness and health literacy, overlap of headache with other medical disorders, and the limited training of neurologists and other physicians managing headache patients on headache diagnosis were frequently reported across the three groups without statistically significant differences (Table 4).

Regarding healthcare system challenges, the lack of specialized headache clinics or trained specialists was reported by all participants in LICs 12/12 (100.0%) and was highly prevalent in LMICs 35/41 (85.4%), but was significantly less common in UMICs 7/20 (35.0%) (*p <* 0.001). Insufficient insurance coverage for investigations and advanced therapies was also a significant challenge, particularly in LICs 11/12 (91.7%) and LMICs 37/41 (90.2%) compared to UMICs 13/20 (65.0%) (*p =* 0.031) (Table 4).

Other commonly reported barriers, including poor patient adherence, overuse of over-the-counter analgesics, high rates of psychiatric comorbidities, use of unregulated complementary therapies, and absence of local or regional guidelines, were prevalent across all three groups without statistically significant differences (Table 4).

## Discussion

This study tried to provide a comprehensive clinician-based assessments of headache care services to date across African countries and highlighted major disparities in healthcare infrastructure, resource availability, and system readiness in the face of the growing burden of headache disorders.

Our results show marked disparities in neurologist density across income categories, with low-income countries reporting the lowest number of neurologists, which significantly contrasts with the high prevalence of headache disorders reported across African countries (2, 3).

Although this discrepancy is well-noted globally due to a shortage in the neurology workforce (17, 18), even in high-income countries. The situation in Africa, especially Sub-Saharan Africa (SSA), could be much more challenging; the majority of African countries (36 countries) have no more than 30 neurologists per country, while 10 countries do not have any neurologists (7). The mismatch between disease burden and specialist availability likely contributes to delayed diagnosis, misclassification of headache disorders, and suboptimal long-term management.

Our results showed that general practitioners and internal medicine physicians were heavily involved in diagnosing and treating headaches in almost all countries; this is indeed an expected consequence of the lack of trained neurologists and paucity of specialized headache centers even at the tertiary care level. This highlights the necessity of standardized headache training programs for these categories of healthcare providers to fill the gap. In this context, many educational interventions have been shown to be beneficial in LMIC settings; for example, the Disease Relief Through Excellent and Advanced Means (DREAM) health program created an initiative to educate more than 10,000 African personnel, including clinical officers, doctors, and nurses, in 11 countries in SSA on how to manage communicable and non-communicable diseases, including headaches from primary and secondary causes. This initiative allowed more patients to be evaluated and receive treatment for their headaches without needing to be seen by a specialist (19).

Recently, through its Regional Outreach Program (ROPE), the IHS partnered with the DREAM program to deliver two structured, in-person training courses to local clinicians in Blantyre, Malawi (in 2022 and 2025). These courses provided primary care providers with the essential skills in the diagnosis and management of primary and secondary headaches. The program also included local mentorship periods and a ‘train-the-trainer’ model. The training output highlighted that partnerships between international societies, such as the IHS, and established local healthcare programs, like DREAM, could pave the way for effective task shifting among primary healthcare providers (20).

Our survey showed that the recently published IHS practice recommendations were the most frequently used in the included countries; this could be attributed to the nicely adopted approach of two-tiered sets of recommendations (optimal and essential) that help cover a wide spectrum of healthcare settings (16, 21). However, the absence of region-specific or country-specific headache management guidelines limits the standardization of care and adaptation to local contexts.

Our data revealed that the access to essential diagnostic tools, particularly CT and MRI, remains markedly limited, especially in LICs. Limited availability of imaging devices, along with high out-of-pocket costs, deficient insurance systems, and the lack of government support or a coordinated national health framework poses challenges to accurate diagnosis, especially in countries where secondary headaches, which mandate brain imaging, are relatively prevalent due to endemic infections, vascular disorders, and traumatic causes (22). These findings are aligned with previous reports from sub-Saharan African countries confirming that imaging facilities remain prohibitively expensive or geographically inaccessible for the majority of the patients (23).

Our findings showed that reimbursed migraine medications are more widely available in LMICs and UMICs as compared to LICs, particularly essential medications [as defined by WHO (World Health Organization) and IHS practice recommendations] such as diclofenac and antiemetics, likely due to their inclusion in the list of essential medicines by WHO (24). Access to sumatriptan remains inconsistent, while newer agents (gepants, ditans, and CGRP monoclonal antibodies) are neither available nor reimbursed in vast majority of the included countries, preventive therapies rely mainly on conventional options like antidepressants and beta blockers. This reflects a prioritization of cost-effective treatments over newer, high-cost innovations due to financial reimbursement constraints. Interventional therapies such as Onabotulinumtoxin A and nerve blocks were significantly more available in LMICs and UMICs compared to LICs. This indicates better resource allocation, greater specialist density, and more developed private healthcare sectors (25).

Supportive therapies, including physiotherapy, psychological support, and nutritional counseling, were reported to be available across LICs, LMICs, and UMICs, with no statistically significant differences between groups. This finding suggests that supportive and non-pharmacological approaches may be more accessible across different healthcare settings compared with some advanced pharmacological or interventional therapies. The relatively widespread availability of these interventions may reflect their lower infrastructure requirements and their potential integration into multidisciplinary headache care models. Psychological support refers to structured interventions such as cognitive behavioral therapy (CBT), biofeedback, relaxation training, stress-management strategies, and psychoeducation. These services aim to address comorbid anxiety, depression, and stress, and to improve coping and quality of life among patients with headache disorders. Nutritional counseling refers to dietary guidance provided to headache patients by healthcare professionals (neurologists, physicians, or dietitians when available). Its role was to help patients identify potential dietary triggers, encourage healthy eating patterns, and support lifestyle modifications relevant to headache care.

The present findings highlight a complex interplay of patient-related, structural, and healthcare system factors contributing to delays in headache diagnosis across different income settings. Self-medication with analgesics, reported universally across all groups, underscores a pervasive global challenge that delays healthcare seeking and contributes to medication-overuse headache. This behavior is likely driven by the over-the-counter availability of analgesics and reflects gaps in patient education and healthcare accessibility. In parallel, cultural beliefs attributing headaches to stress or non-medical causes were commonly reported, reinforcing the influence of sociocultural context on health-seeking behavior. The significantly higher reliance on traditional remedies in LICs and LMICs further illustrates how cultural practices and limited access to formal healthcare systems, potentially delaying timely diagnosis and appropriate management (9).

Misinformation from social media and online sources emerged as a notable contributor, particularly in middle-income countries, suggesting that increased digital access does not necessarily translate into improved health literacy. Instead, it may expose patients to inaccurate or misleading information, shaping inappropriate self-management strategies. These findings emphasize the need for targeted public health interventions to improve digital health literacy and promote reliable sources of information (26).

Structural barriers were markedly more pronounced in LIC, where prolonged waiting times and limited access to neuroimaging were significantly more common. These factors reflect systemic healthcare limitations, including inadequate infrastructure, workforce shortages, and insufficient resource allocation. The high burden of out-of-pocket costs and limited insurance coverage further exacerbate inequities in access to care, potentially leading to delayed diagnosis and poorer outcomes (9).

Healthcare system challenges also played a critical role, particularly the lack of specialized headache clinics and trained professionals, which was universally reported in LICs. This shortage of expertise may contribute to under diagnosis of headache disorders. Additionally, insufficient insurance coverage for advanced investigations and therapies in LICs and LMICs highlights ongoing gaps in comprehensive headache care (9).

Importantly, several barriers, including limited awareness, poor adherence to medications, the widespread use of unregulated complementary therapies, inadequate training in headache diagnosis, and absence of local or regional guidelines, were consistently reported across all income groups. This suggests that, beyond resource constraints, there are fundamental gaps in education, training, and guideline implementation that transcend economic differences. Collectively, these findings underscore the need for multifaceted strategies that address both system-level deficiencies and patient-related factors (27).

A wider context regarding healthcare provision in Africa implies the need to consider the systemic challenges hindering optimal care. The civil wars, violence, conflicts, and political tensions in several parts of Africa affected healthcare systems and caused loss of human resources, destruction of facilities, and disruption of essential health care services (9).

This study has several notable strengths. First, it provides a multinational perspective by including neurologists from LICs, LMICs, and UMICs, enabling meaningful comparisons across diverse healthcare systems and highlighting important inequities in headache care. Second, the inclusion of underrepresented countries with limited published data, addresses a critical gap in literature.

There are still several limitations with this study. Although stratified sampling was employed to ensure representation across income strata, participation ultimately depended on clinician availability and willingness to respond. This introduced potential selection bias. Additionally, despite the diverse geography, it may not fully represent all African countries. Furthermore, the findings reflect clinicians’ perspectives and may not fully capture patient experiences or outcomes.

A noteworthy methodological limitation of this study is that neurologist workforce size was analyzed as absolute numbers reported by clinicians, without normalization by population size. Although normalization (e.g., neurologists per million inhabitants) would provide a more precise measure of workforce density and allow more meaningful cross-country comparisons, this was not feasible because reliable, up-to-date population denominators and neurologist density figures were not consistently available across all included countries at the time of data collection. Consequently, the reported figures should be interpreted as indicative of relative workforce capacity rather than exact density estimates, and caution is warranted when extrapolating these results to population-level needs.

A key limitation of this study is that workforce capacity data were derived from clinician self-reports, without systematic verification against official registries. Although participants were instructed to consult relevant authorities when possible, this could not be mandated, and therefore the data may be subject to information bias. As such, our findings should be interpreted as reflecting clinician perceptions and indicative trends rather than definitive national statistics. This limitation underscores the need for future studies to integrate registry-based data with clinician surveys to provide a more validated picture of headache care capacity across African countries.

Another limitation of this study is that the survey assessed availability of elective neurological consultation but did not capture emergency support or urgent consultation pathways. As a result, the findings may underestimate disparities in timely access to acute headache care.

Finally, the fixed survey design restricted flexibility to capture contextual nuances of clinical practice, limiting exploration of issues beyond the predefined questions. In addition, the survey did not collect detailed information on participants’ workplace settings or practice environments.

## Conclusion

This study, based on a survey of neurologists with recognized expertise in headache medicine across multiple African countries, highlights substantial disparities in workforce capacity, access to essential medications, and the health system infrastructure required for effective headache care. Addressing these challenges requires coordinated, context-customized strategies involving healthcare authorities, policymakers, academic entities, and patient societies. Strengthening headache care systems on the continent of Africa is essential to reduce the substantial burden associated with headache disorders and improve neurological health outcomes.

## Data Availability

The raw data supporting the conclusions of this article will be made available by the authors, without undue reservation.

## References

[1] Stovner LJ Hagen K Linde M Steiner TJ. The global prevalence of headache: an update, with analysis of the influences of methodological factors on prevalence estimates. J Headache Pain. (2022) 23:34. doi: 10.1186/s10194-022-01402-2, 35410119 PMC9004186

[2] Adoukonou T Agbetou M Dettin E Kossi O Husøy A Houinato D . The burden of headache disorders in Benin: national estimates from a population-based door-to-door survey. J Headache Pain. (2025) 26:56. doi: 10.1186/s10194-025-01992-7, 40098152 PMC11912794

[3] Maiga Y Diallo SH Sangho O Moskatel LS Konipo F Bocoum A . The burden of headache and a health-care needs assessment in the adult population of Mali: a cross-sectional population-based study. J Headache Pain. (2024) 25:107. doi: 10.1186/s10194-024-01811-5, 38937699 PMC11212246

[4] Kuate Tegueu C Dzudie Tamdja A Kom F Forgwa Barche B Ebasone P Magnerou M . Headache in the adult population of Cameroon: prevalence estimates and demographic associations from a cross-sectional nationwide population-based study. J Headache Pain. (2024) 25:42. doi: 10.1186/s10194-024-01748-9, 38515027 PMC10956204

[5] Mbewe E Zairemthiama P Yeh HH Paul R Birbeck GL Steiner TJ. The epidemiology of primary headache disorders in Zambia: a population-based door-to-door survey. J Headache Pain. (2015) 16:515. doi: 10.1186/s10194-015-0515-7, 25916334 PMC4401479

[6] Zebenigus M Tekle-Haimanot R Worku DK Thomas H Steiner TJ. The prevalence of primary headache disorders in Ethiopia. J Headache Pain. (2016) 17:110. doi: 10.1186/s10194-016-0704-z, 27924616 PMC5142157

[7] Kissani N Liqali L Hakimi K Mugumbate J Daniel GM Ibrahim EAA . Why does Africa have the lowest number of neurologists and how to cover the gap? J Neurol Sci. (2022) 434:120119. doi: 10.1016/j.jns.2021.120119, 34982975

[8] Puledda F de Boer I Messina R Garcia-Azorin D Portes Souza MN Al-Karagholi MA . Worldwide availability of medications for migraine and tension-type headache: a survey of the international headache society. Cephalalgia. (2024) 44:3331024241297688. doi: 10.1177/03331024241297688, 39552307

[9] Mortel D Kawatu N Steiner TJ Saylor D. Barriers to headache care in low- and middle-income countries. eNeurologicalSci. (2022) 29:100427. doi: 10.1016/j.ensci.2022.100427, 36212617 PMC9539775

[10] Mateen FJ Dua T Steiner T Saxena S. Headache disorders in developing countries: research over the past decade. Cephalalgia. (2008) 28:1107–14. doi: 10.1111/j.1468-2982.2008.01681.x, 18727634

[11] Raffaelli B Rubio-Beltrán E Cho S-J De Icco R Labastida-Ramirez A Onan D . Health equity, care access and quality in headache – part 2. J Headache Pain. (2023) 24:167. doi: 10.1186/s10194-023-01699-7, 38087219 PMC10717448

[12] von Elm E Altman DG Egger M Pocock SJ Gøtzsche PC Vandenbroucke JP. The strengthening the reporting of observational studies in epidemiology (STROBE) statement: guidelines for reporting observational studies. J Clin Epidemiol. (2008) 61:344–9. doi: 10.1016/j.jclinepi.2007.11.008, 18313558

[13] World Bank. World Bank Country and Lending Groups. Washington, DC: World Bank (2025).

[14] Eysenbach G. Improving the quality of web surveys: the checklist for reporting results of internet E-surveys (CHERRIES). J Med Internet Res. (2004) 6:e34. doi: 10.2196/jmir.6.3.e34, 15471760 PMC1550605

[15] Google LLC. Healthcare Service Provided for Headache Patients in Selected African Countries: A Cross-sectional Survey of Clinician Perspectives. Menlo Park, CA: Google LLC (2025). 10.3389/fneur.2026.1853307 PMC13414887 42528641

[16] Puledda F Sacco S Diener HC Ashina M Al-Khazali HM Ashina S . International headache society global practice recommendations for preventive pharmacological treatment of migraine. Cephalalgia. (2024) 44:3331024241269735. doi: 10.1177/03331024241269735, 39262214

[17] Di Liberto G Baldizzi G Carvalho V Cuffaro L Sauerbier A Klingelhoefer L . Education research: impact of burnout on neurology residents and research fellows in Europe. Neurol Educ. (2022) 1:e200035. doi: 10.1212/NE9.0000000000200035, 40809503 PMC12339222

[18] World Health Organization. Optimizing Brain Health Across the Life Course: WHO Position Paper. Geneva: World Health Organization (2022).

[19] Leone M Palombi L Guidotti G Ciccacci F Lunghi R Orlando S . What headache services in sub-Saharan Africa? The DREAM program as possible model. Cephalalgia. (2019) 39:1339–40. doi: 10.1177/0333102419849014, 31072131

[20] Martinelli D Leone M Dodd-Glover F Tassorelli C. The regional outreach Programme of the international headache society: a WHO IGAP-oriented initiative in partnership with DREAM to improve healthcare for people with headache in sub-Saharan Africa. Cephalalgia Rep. (2025) 8:25158163251396915. doi: 10.1177/25158163251396915 41335012

[21] Puledda F Sacco S Diener HC Ashina M Al-Khazali HM Ashina S . International headache society global practice recommendations for the acute pharmacological treatment of migraine. Cephalalgia. (2024) 44:3331024241252666. doi: 10.1177/03331024241252666, 39133176

[22] Genc H Baykan B Bolay H Uluduz D Unal-Cevik I Kissani N . Cross-sectional, hospital-based analysis of headache types using ICHD-3 criteria in the Middle East, Asia, and Africa: the head-MENAA study. J Headache Pain. (2023) 24:24. doi: 10.1186/s10194-023-01555-8, 36915115 PMC10010217

[23] Onwuchekwa CR Onwuchekwa AC. The role of computed tomography in the diagnostic work-up of headache patients in Nigeria. Headache. (2010) 50:1346–52. doi: 10.1111/j.1526-4610.2010.01712.x, 20572879

[24] World Health Organization. Expert committee on selection and use of essential medicines. Geneva: World Health Organization (2026).

[25] Steiner TJ. Lifting the burden: the global campaign against headache. Lancet Neurol. (2004) 3:204–5. doi: 10.1016/S1474-4422(04)00703-3, 15039030

[26] Eysenbach G. Infodemiology and infoveillance tracking online health information and cyberbehavior for public health. Am J Prev Med. (2011) 40:S154–8. doi: 10.1016/j.amepre.2011.02.006, 21521589

[27] Steiner TJ Jensen R Katsarava Z Linde M MacGregor EA Osipova V . Aids to management of headache disorders in primary care (2nd edition): on behalf of the European headache federation and lifting the burden: the global campaign against headache. J Headache Pain. (2019) 20:57. doi: 10.1186/s10194-018-0899-2, 31113373 PMC6734476

