# Supplementary material for: Healthcare service provided for headache patients in selected African countries: a cross-sectional survey of clinician perspectives
Source: Front Neurol. 2026 Jul 15;17:1853307. doi: 10.3389/fneur.2026.1853307 (PMC13414887; doi:10.3389/fneur.2026.1853307)
Supplement: Supplementary file 1 [file Table_1.DOCX]

**Healthcare Service Provided for Headache Patients in Selected African Countries: A Cross-sectional Survey of Clinician Perspectives**

1. **Age**
2. **Sex**
3. **Country**
4. **Workplace**

- General hospital
- Insurance hospital
- University hospital
- Private hospital
- Private clinic
- Primary healthcare center

1. **Years of experience in Headache**
2. **The estimated number of Neurologists in your country**
3. **The estimated number of headache clinics in your country**
   - 0
   - 1–5
   - 6–10
   - 11–15
   - More than 15
4. **Do the following providers diagnose/treat headaches? ***
   - General practitioners
   - Internal medicine doctors
   - Nurses
   - Pharmacists
5. **Medical guidelines followed in headache management**
   - IHS
   - EHF
   - AHS
   - NICE
   - Local/Regional
   - Others (please specify)
6. **Average waiting list for headache consultation**
   - No waiting list
   - <1 week
   - 1–2 weeks
   - 3–4 weeks
   - 5–6 weeks
   - 7–8 weeks
   - > 8 weeks
7. **Average waiting list for investigations**
   - No waiting list
   - <1 week
   - 1–2 weeks
   - 3–4 weeks
   - 5–6 weeks
   - 7–8 weeks
   - > 8 weeks
8. **Type of Telemedicine available for headache patients**
   - Local
   - Regional
   - International
9. **Reimbursed acute migraine medications**
   - Paracetamol
   - Aspirin
   - Ibuprofen
   - Naproxen
   - Diclofenac
   - Indomethacin
   - Sumatriptan
   - Rizatriptan
   - Zolmitriptan
   - Naratriptan
   - Almotriptan
   - Eletriptan
   - Frovatriptan
   - Ergotamine tartrate
   - Dihydroergotamine
   - Metoclopramide
   - Domperidone
   - Prochlorperazine
   - Chlorpromazine
   - Ubrogepant
   - Rimegepant
   - Zavegepant
   - Lasmiditan
   - Others (please specify)
10. **Reimbursed preventive migraine medications**
    - Propranolol
    - Metoprolol
    - Atenolol
    - Nadolol
    - Amitriptyline
    - Nortriptyline
    - Venlafaxine
    - Valproic acid
    - Topiramate
    - Gabapentin
    - Verapamil
    - Flunarizine
    - Cinnarizine
    - Erenumab
    - Fremanezumab
    - Galcanezumab
    - Eptinezumab
    - Others (please specify)
11. **Available interventional pain therapy for headache patients**
    - Onabotulinumtoxin A
    - Nerve block
    - Radiofrequency
    - Neuromodulation
    - Others (please specify)
12. **Available supportive therapy for headache patients**

- Psychological support
- Physiotherapy
- Nutritional counseling
- Others (please specify)

1. **The reasons for the delay in diagnosis in some headache cases**
   - Cultural beliefs often attribute headaches to stress, spirits, or lifestyle rather than a neurological disorder
   - Self-medication with analgesics
   - Use of traditional remedies
   - Limited awareness and health literacy
   - Wrong information from social media or online sources
   - Long waiting list to receive consultation with a headache specialist or to undergo investigations.
   - Limited access to neuroimaging
   - Out-of-pocket costs and lack of insurance
   - Overlap with other medical disorders
   - Limited training in headache diagnosis
   - Others (please specify)
2. **The main challenges in your country in providing good care for headache patients**
   - Lack of specialized headache clinics or headache specialists
   - Insufficient insurance coverage for investigations and advanced therapies.
   - Unavailability of newer therapies
   - Poor adherence of the patients to the prescribed medications
   - Overuse of over-the-counter analgesics leads to medication-overuse headache
   - High prevalence of comorbid anxiety, depression, or sleep disorders
   - Use of unregulated complementary or alternative treatments without medical guidance
   - Absence of local or regional guidelines tailored to African healthcare systems
   - Others (please specify)
